# Topics of Public Interest

**Published:** 1914-05

**Authors:** 


### TOPICS OF PUBLIC INTEREST

**The Syracuse University Medical College** celebrated the opening of the new Dispensary March 30, 1914. During the afternoon the building was open for inspection. It is the consensus of expert opinion that this new Dispensary Building is as nearly perfect as careful study of dispensary needs, architectural ability and skilled workmanship can combine to make. It has especially designed rooms to accommodate the following clinics: On the first floor, Obstetrics and Gynecology, Pediatrics, Genito-Urinary Diseases, Orthopedics, and Tuberculosis with the pharmacy and offices for clerks, medical superintendent and staff. On the second floor, General Surgery, Proctology, Nose, Throat and Ear, Dermatology and Syphilis, Internal Medicine, Psychiatry and Neurology, and Ophthalmology. In the basement are the X-Ray room, an orthopedic shop, a milk-distributing station and the manufacturing pharmacy. The third floor is occupied by the laboratories for chemical and microscopical diagnosis, bacteriology, and research work. A small laboratory for instant use is provided with each large clinic. The furniture and equipment is of the latest model.

In the evening a public meeting was held at which Chancellor Day formally presented the use of the building and equipment to the Syracuse Free Dispensary Association, which will continue the conduct of the Dispensary as a charity, while vesting in the faculty of the College of Medicine the organization and administration of the medical and surgical service. The address of the evening was made by Dr. Richard C. Cabot of the Harvard Medical School, who with his characteristic forcefulness impressed upon the audience the importance of dispensary work, and of the great advances in medical practice which would be gained by team work.

---

**Early Campaign Against Flies.** The Merchants' Assn. of New York City has issued a card emphasizing the importance of a fly campaign before these pests become especially troublesome. It is estimated that the progeny of one pair of flies—not to imply that flies are monogamic—would, in a season, if all lived, fill a space equal to the Woolworth Building. Moreover, flies are more easily captured before the weather becomes warm. The passing of the horse has removed thousands of manure boxes which formerly bred flies.

---

**IOLA SANITARIUM.** The Board of Supervisors of Monroe Co. recently appropriated $75,000 for enlargement and maintenance of this splendid institution. At a special meeting March 20 the various medical societies of Rochester and Monroe Co. made formal application for a further appropriation of $50,000. This would represent a per capita tax of nearly half a dollar for the one purpose. It is worth it. But, we recently computed that, including public education, but not including the general maintenance of parks, public institutions, the health department and other enterprises which, in theory at least, contribute to the welfare of taxpayers themselves, over a quarter of the total tax for Buffalo is for strictly benevolent and philanthropic purposes. This is probably typic of other cities. It seems to us an open question whether benevolence should be compulsory and, at least, it is only fair that the taxpayer should count the part of his tax paid for benevolence in estimating his further, voluntary contributions.

---

**Deaths from Intra-Spinal Injection of Serum containing Neo-Salvarsan.** Early in March, eight patients were treated at the County Hospital of Los Angeles, with fatal results. All were proved syphilitic by Wassermann and butyric acid tests, the diagnosis being confirmed by necropsy. On account of reports of failure of salvarsan intravenously, it was decided to inject intraspinously, serum, drawn from the patients and used as a solvent, along with physiologic salt solution, for neosalvarsan. Thorough asepsis was employed for the whole procedure. Death resulted within two or three days. It is suggested that oxidation had occurred in the drug.

---

**Automobile Regulations for Buffalo.** Contrary to expectation, the aldermen have exempted slowly moving vehicles from carrying lights, and have refused the privilege of standing on lower Main street. On most other down-town streets automobiles may stand an hour unattended.

---

**Heroine-Cocaine Bill.** The Frawley bill, awaiting the governor's signature, places heroine under the same restrictions as cocaine. The physician may not keep more than 1⅛ ounces on hand . A book must be kept for entering purchases and, in it, the place in which the drug is stored, must be recorded.

---

**Bichloride of Mercury Bill.** Awaiting the governor's signature, the bill is effective June 1, 1914, and provides that the drug must not be sold or given away without a physician's prescription, and that, if dispensed in tablets, they must be colored green and of coffin shape. This shape has been patented and the patent deeded to the American Pharmaceutical Assn., to prevent monopoly.

---

**Boylan Narcotic Bill.** Awaiting the governor's signature, this applies especially to chloral and opium and derivatives. A physician's, veterinarian's or dentist's prescription is required, except for an exemption of domestic remedies containing only small amounts. The prescriber must give his full name, address, office hours and telephone, and the name, age and address of the patient, with date. The maximum content of one prescription is 4 grains of morphine, two of heroine (note the inconsistency), six of codeine or 4 drachms of chloral, unless the dispenser verifies the prescription by consultation with the prescriber by telephone or otherwise. Hypodermic syringes and needles cannot be sold except on prescription, and the name and address of the purchaser must be recorded as now required in N. Y. City: Records of prescriptions must be kept for five years. The constant use of any habit-forming drug, except under the direction of a physician, renders the victims supject to commitment to a public or private licensed institution. Physicians addicted to the use of such drugs are liable to revocation of license, subject to restoration after cure.

---

**Tuberculosis Mortality.** There has been a decline from 262 per 100,000 population in 1896 to 166 in 1912, for New York City; from 132 in 1897 to 114 in 1912 for the rest of the state. In spite of the increase of population, there has been an almost regular decrease in the absolute number of deaths from 4158 in 1907 to 3826 in 1913, in the non-Metropolitan part of the state. It certainly seems that the movement for tuberculosis hospitals and the general education and enforcement of sanitary and hygienic principles is bearing good fruit. It should be borne in mind that this decrease is genuine; it is too marked and regular to be accidental, and there has been no increase in other diseases that might be regarded as removing a part of the population otherwise destined to die of tuberculosis. On the contrary, the mortality from other diseases has diminished.

---

**Harrington Lectures.** The Uuiversity of Buffalo announces that Prof. Ludwig Pick of Berlin—whose work on "frosted" liver (Zuckergussleber) is especially noteworthy—will deliver this course at the Commencement season, the title being Some Advances in Pathologic Anatomy.

---

**Race Distribution of Population of Buffalo.** German 26%; English and Celtic 21%; Polish 15%; Italian 5%; Hebrew 1.6%; French 1.2%; total foreign 72%. These proportions undoubtedly apply to direct and one-generation foreignness. It is scarcely conceivable that any northern American city has 28% of colonial American stock.

---

**Milk Prices.** The Bordens Condensories announce the following schedule per hundredweight: April, $1.30; May, $1.15; June, $1.05; July, $1.15; August, $1.25; September, $1.40. An additional 10 cents per hundredweight will be paid for milk testing 3.8% of butter fat. It will be noted that these prices correspond to about 2½ cents a quart. They are prices at which it pays the farmers in grazing districts to produce milk. It certainly seems that, by condensation and preparation of milk into powder form, the cost to the ultimate consumer can be kept down.

---

**Social Service for Paroled Insane.** This has been inaugurated by the State Charities Aid Assn. The State Hospitals, with 32,600 inmates, have become overcrowded so that a law has been passed providing for the establishment of out-departments and special observation of paroled patients. It is believed that the suspicion felt regarding trivial manifestation of emotions and the misconstruction of the actions of paroled patients often produces relapse, and that kindly supervision will enable many to remain at large who would otherwise be forced back into hospitals. On the other hand, the parole of patients is not without danger. We recall the case of an old woman who had been returned to her family. She seemed to be quite sane, and was often trusted alone with children. On one occasion she told her family never to leave her with children again, as, several times, she had been on the point of killing them. Patients often have a realization of their need of restraint. An amusing story which may be true, is told illustrative of this. A man seemed to have recovered his sanity and was allowed to write a letter home, announcing his cure. After finishing the letter and directing the envelope, he licked a stamp and accidentally dropped it. By chance it fell on a cockroach. The patient, amazed, saw the stamp moving across the floor, tore up the letter and resigned himself to a longer stay in the asylum.

---

**Death from Typhoid After Vaccination.** The Board of Health of Queens County has officially declared the cause of death in the case of Clarence Panza, to be typhoid. Three weeks previously, he had received the final inoculation against typhoid. This is said to be the first death from typhoid occurring in the military experience of this country—the deceased being a member of the hospital corps of the 13th coast artillery district, N. G. N. Y.,—in one who had been inoculated. Although the lapse of time after even the final inoculation renders it improbable that the typhoid was due to this method, its failure is important.

---

**Size of French Families.** Of eleven million families, two were childless; 3 had one child each; 2½ had 2; 1½ had 3; 1 had 4; ½ million had 5; ½ million had 6 or more. There were 34 families with 17 children, and 45 with 18 each. Owing to the inevitable high mortality in infancy and, indeed, up to adolescence, sterility, celibacy, etc., it has been estimated that the average couple must have four children in order to keep the population stationary. Probably, under present conditions, and especially allowing for the greater care of children in small families, an average of four children would result in a slight progressive increase.

---

**The Buffalo Academy of Medicine** has purchased a lot on Linwood avenue near North street, and expects to build a club-house in the comparatively near future.

---

**Medical Judges.** Senator J. L. Seeley has introduced a bill in the New York legislature, providing for a medical judge, at a salary of $7,500, and an allowance of $2,500 for traveling expenses, for each district of the Appellate Court. He is to attend every trial at which medical evidence is given as to sanity, and to furnish expert advice to court and jury. His instructions are to have equal weight with those of the trial judges.

---

**Aviation Mortality.** For the first three months of 1914, 36 aviators were killed. The total mortality to April 1, 1914 is 462.

---

**Failure of Transfusion.** The wife of Dr. W. W. Percy of Rochester died April 10, of acute pernicious anaemia, in spite of transfusion from her husband and from her brother, one quart of blood being taken from each.

---

**Typhoid at Bridgeburg.** Dr. Collins, Health Officer, has encountered four cases, in M. C. R. R. employees. This road has a special intake from Niagara River below the village intake.

---

Water as a Carbon Remover. While the motor is hot and operating fast, introduce 100 c.c. of water through the air valve. This breaks up the scales. Follow immediately with kerosene, through the primer or air intake of carburetor. Repeat about every thousand miles.

---

Cervical Rib. N. Gilbert, *Am. Jour. of Med. Sci.,* Sept., 1913, reports seven cases with general remarks. He considers the X-ray the only certain method of diagnosis.

